# Transverse Testicular Ectopia: A Rare Presentation with Persistent Müllerian Duct Syndrome

**DOI:** 10.4274/jcrpe.1479

**Published:** 2014-09-05

**Authors:** Onur Telli, Mehmet İlker Gökçe, Perviz Haciyev, Tarkan Soygür, Berk Burgu

**Affiliations:** 1 Ankara University, Faculty of Medicine, Department of Pediatric Urology, Ankara, Turkey; 2 Ankara University, Faculty of Medicine, Department of Urology, Ankara, Turkey

**Keywords:** Orchiopexy, persistent Müllerian duct syndrome, testis, transverse testicular ectopia

## Abstract

Undescended testes can be found in the inguinal channel or in the abdomen. Rarely, undescended testes can present with transverse testicular ectopia (TTE) and very rarely, with residual Müllerian duct (MD) structures. This latter presentation is called persistent MD syndrome (PMDS). PMDS is mostly discovered during surgery for inguinal hernia or cryptorchidism. TTE is a rare congenital anomaly in which both testes descend through a single inguinal canal. Patients with TTE present with symptoms of unilateral cryptorchidism and contralateral inguinal hernia. Herein, we report two TTE cases: one associated with PMDS and the other having only cross ectopia. For patients with inguinal hernia and cryptorchidism associated with TTE, PMDS should be kept in mind and radiologic evaluation with ultrasonography or magnetic resonance imaging of the genitourinary system and karyotyping are recommended. Radiologic evaluation can be helpful in the diagnosis of TTE; however, it cannot diagnose the malignancy itself. Laparoscopy is very useful for both diagnosis and treatment of TTE.

## INTRODUCTION

Transverse testicular ectopia (TTE) is an extremely rare anomaly in which both testes descend through the same inguinal canal into the same hemiscrotum and is usually associated with an inguinal hernia. Most patients with TTE present with contralateral inguinal hernia and a unilateral cryptorchidism. Many different nomenclatures such as crossed testicular ectopia, testicular pseudoduplication, unilateral double testes and transverse aberrant testicular maldescent have been used for this condition (1). TTE is associated with persistent Müllerian duct syndrome (PMDS) in approximately 20% of the cases (2). In PMDS, MD derivatives such as the uterus, fallopian tubes and the upper two-thirds of a vagina are present in otherwise normal, virilized males (karyotype 46XY). The Müllerian system usually regresses in males, but occasionally, residual Müllerian structures exist. These well-developed MD derivatives may be localized intra-abdominally or may herniate in the inguinal region (3).

Herein, we report our experience with two cases of TTE: one who was discovered during laparoscopic exploration for bilateral undescended testes with cross ectopia and the other-during inguinal exploration for a left palpable testis in the inguinal canal in a patient with PMDS.

## CASE REPORT

**Case 1**

**Figure 1:**
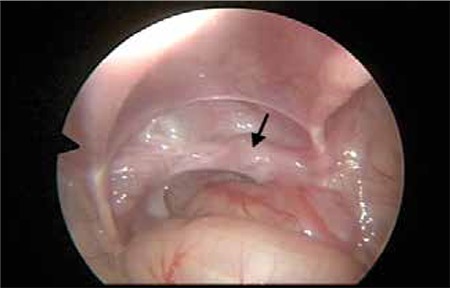
Laparoscopic visualization of left transverse testicular ectopia (Arrow shows the left testis =TTE)

An 8-month-old boy was referred with bilateral impalpable undescended testis. Diagnostic laparoscopy was performed initially which revealed that the left spermatic vessels and the vas deferens ran toward the right internal inguinal ring. The right testis was located just cephalad to the internal inguinal ring. On careful inspection, the two testes and testicular structures were observed on the antero-superior side of the bladder. No MD remnant was found. The testes were connected by a common vas deferens. The right spermatic cord was entering the right internal inguinal ring and the left testis had migrated to the right pulling the spermatic cord externally (crossed testicular ectopia) (Figure 1). Using laparoscopy, the crossing vas and vessels were carefully dissected after incising the covering peritoneum. Both testes were brought down through the right/left inguinal canal and each was placed on its respective side of the scrotum. Karyotyping showed a male XY pattern. Throughout the 3-month follow-up, the size and blood flow of the testes were normal as assessed by ultrasound.

**Case 2**

**Figure 2:**
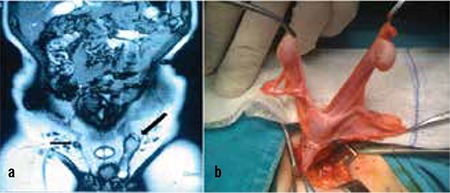
MR images of both inguinal canals, showing right atrophic and left normal testes located in the corresponding inguinal canalb) Intraoperative view of the testes, uterus and fallopian tubes in the left inguinal canal

The second case was an 18-month-old boy who was referred to our clinic for evaluation of bilateral impalpable testis. On clinical examination, we found that the right testis was nonpalpable and the left one was palpable in the inguinal canal. Magnetic resonance imaging revealed a relatively small right and a larger left testis, both located in the inguinal canals (Figure 2a). Laparoscopy for the nonpalpable right testis and left inguinal exploration was carried out to understand the anatomy of the right spermatic cord as well as to help us in the management of the patient and in the decision for bilateral orchiopexy. We found his right spermatic cord and vessels in the left internal inguinal canal from the right side suggesting the presence of a transverse testicular ectopia. When the processus vaginalis was opened, a primitive uterus-like structure, together with fallopian tubes, was identified and excised (Figure 2b).

We carried out orchiopexy for both testes to their corresponding sites of the scrotum. A primitive dysgenetic uterus was reported after postoperative histologic examination. The karyotype confirmed a male gender (46XY). One year after the operation, ultrasonography showed both testes to be in a good condition.

## DISCUSSION

TTE, also named testicular pseudo-duplication, is characterized by both testes descending through a single inguinal canal. The precise etiology of TTE is still unclear. Various anatomic factors (defective implantation, rupture or tearing of the gubernaculum, obstruction of the internal inguinal ring, development of adhesions between the testis and adjacent structures, late closure of the umbilical ring, etc.) are suggested as causative or inducible factors in failure of testicular descent (4). Some of TTE cases are very rarely associated with PMDS, which is caused by defects in the synthesis or action of Müllerian-inhibiting factor and is characterized by karyotypically normal males with retained MD derivatives. PMDS is mostly discovered during surgery for inguinal hernia or for cryptorchidism (5). TTE also helps in the identification of PMDS, as in our second case. In patients with inguinal hernia and cryptorchidism, possible TTE and PMDS must be considered requiring radiologic evaluation of the genitourinary system and karyotyping. Treatment of PMDS is exclusively surgical and aims to correct cryptorchidism. In PMDS, the testes are usually histologically normal, apart from lesions due to long-standing cryptorchidism. The overall incidence of malignant transformation in these testes is 18%, similar to the rate in abdominal testes in otherwise normal men. There also have been reports of embryonal carcinoma, seminoma, yolk sac tumor and teratoma in patients with PMDS (6). The undescended testes are at increased risk of malignant transformations arising from Müllerian remnants to adenocarcinoma, squamous cell carcinoma and papillary cystadenocarcinoma. Therefore, due to the risk of tumor in the undescended testis, necessary surgical intervention must be made to prevent life-threatening testicular cancers (7).

In conclusion, TTE is a rare condition and should be suspected in patients presenting with inguinal hernia on one side and cryptorchidism on the other side. As PMDS and TTE are usually discovered incidentally during surgery for undescended testis or inguinal hernia, the optimal surgical approach should include testicular biopsies, herniotomy, orchidopexy and excision of MD remnants without risking the vas deferens. A long-term follow-up will be needed for assessment of the fertility in these patients.

## References

[1] Naji H, Peristeris A, Stenman J, Svensson JF, Wester T (2012). Transverse testicular ectopia: three additional cases and a review of the literature. Pediatr Surg Int.

[2] Karnak I, Tanyel FC, Akçören Z, Hiçsönmez A (1997). Transverse testicular ectopia with persistent müllerian duct syndrome. J Pediatr Surg.

[3] Josso N, Belville C, di Clemente N, Picard JY (2005). AMH and AMH receptor defects in persistent Müllerian duct syndrome. Hum Reprod Update.

[4] Josso N, Bierich JL, Rager K Ranke (1977). Development and descent of the fetal testis. Maldescensus testis.

[5] Acikalin MF, Pasaoglu O, Tokar B, Ilgici D, Ilhan H (2004). Persistent Mullerian duct syndrome with transverse testicular ectopia: a case report with literature review. Turk J Med Sci.

[6] Shinmura Y, Yokoi T, Tsutsui Y (2002). A case of clear cell adenocarcinoma of the müllerian duct in persistent müllerian duct syndrome: the first reported case. Am J Surg Pathol.

[7] Farikullah J, Ehtisham S, Nappo S, Patel L, Hennayake S (2012). Persistent Müllerian duct syndrome: lessons learned from managing a series of eight patients over a 10-year period and review of literature regarding malignant risk from the Müllerian remnants. BJU Int.

